# Case report: open surgical management of Bouveret syndrome

**DOI:** 10.1093/jscr/rjae809

**Published:** 2025-04-01

**Authors:** Neha Patchipala, Christina Guyn, Veronica Guerrero

**Affiliations:** Department of General Surgery, Rosalind Franklin University of Medicine and Science, 3333 N Green Bay Rd, North Chicago, IL 60064, United States; Department of General Surgery, Rosalind Franklin University of Medicine and Science, 3333 N Green Bay Rd, North Chicago, IL 60064, United States; Department of General Surgery, Northwestern Medicine, 10400 Haligus Rd, Huntley, IL 60142, United States

**Keywords:** Bouveret syndrome, gallstone ileus, case report, surgical management

## Abstract

Bouveret syndrome is a rare type of gallstone ileus, where a gallstone obstructs the duodenum due to a cholecystoduodenal fistula, accounting for 1–3% of cases, predominantly in women. The presentation of Bouveret syndrome is often nonspecific, including acute cholangitis, nausea, vomiting, and abdominal pain, with significant morbidity and mortality risks. We report a 73-year-old man with complicated gallstone ileus, presenting with abdominal pain, nausea, and vomiting. Imaging revealed a 3-cm stone located in the proximal duodenum and was suspected to be associated with a choledochoenteric fistula. The procedure started laparoscopically but was converted to open surgery, successfully removing the stone. Postoperatively, the patient required bowel rest and total parenteral nutrition. He was discharged with a gastrostomy tube and a Jackson–Pratt drain for outpatient follow-up. A standardized surgical protocol that includes both endoscopic and open techniques should be investigated for effective management of Bouveret syndrome.

## Introduction

Bouveret Syndrome, a rare complication of cholelithiasis, occurs when a gallstone obstructs the gastrointestinal tract, usually the duodenum [1]. Patients present with symptoms of bowel obstruction, including nausea, vomiting, abdominal pain, and distension [2]. Although typically affecting the duodenum, it can also involve the jejunum or ileum, complicating diagnosis. Risk factors include advanced age, female gender, and a history of gallbladder disease, especially postcholecystectomy or in patients with large gallstones [3].

A fistula between the gallbladder and gastrointestinal tract allows gallstones to migrate [4]. Diagnostic tools include abdominal X-rays, CT scans, and endoscopy, with CT being particularly effective for imaging obstructions and fistulas [3]. Management usually involves surgery—enterotomy, stone extraction, and fistula repair [5]. Endoscopic techniques can be utilized if the stone is accessible. Surgical interventions carry morbidity and mortality rates of 37.5% and 11%, respectively [6]. Surgery may be unfeasible for elderly patients with comorbidities [7]. Here, we describe an elderly male with complicated gallstone ileus and acute cholangitis who required open surgical management due to duodenal stone impaction.

## Case presentation

A 73-year-old male with a history of atrial fibrillation, benign hypertension, gout, hyperlipidemia, right carotid stenosis, and cholelithiasis presented to the emergency department with diffuse abdominal pain and nausea/vomiting lasting 2 days. He reported discomfort in the epigastric region, with eating exacerbating his symptoms. The patient denied fever, chills, cough, chest pain, or diarrhea. His medical history included bladder surgery but no previous abdominal surgeries.

His vital signs were blood pressure 153/97 mmHg, pulse 70 bpm, respiratory rate 22 breaths/min, temperature 99.5°F, and oxygen saturation 95% on room air. He showed no acute distress or sweating. The abdominal exam revealed distension with a soft abdomen and mild tenderness but no guarding or rebound tenderness.

**Figure 1:**
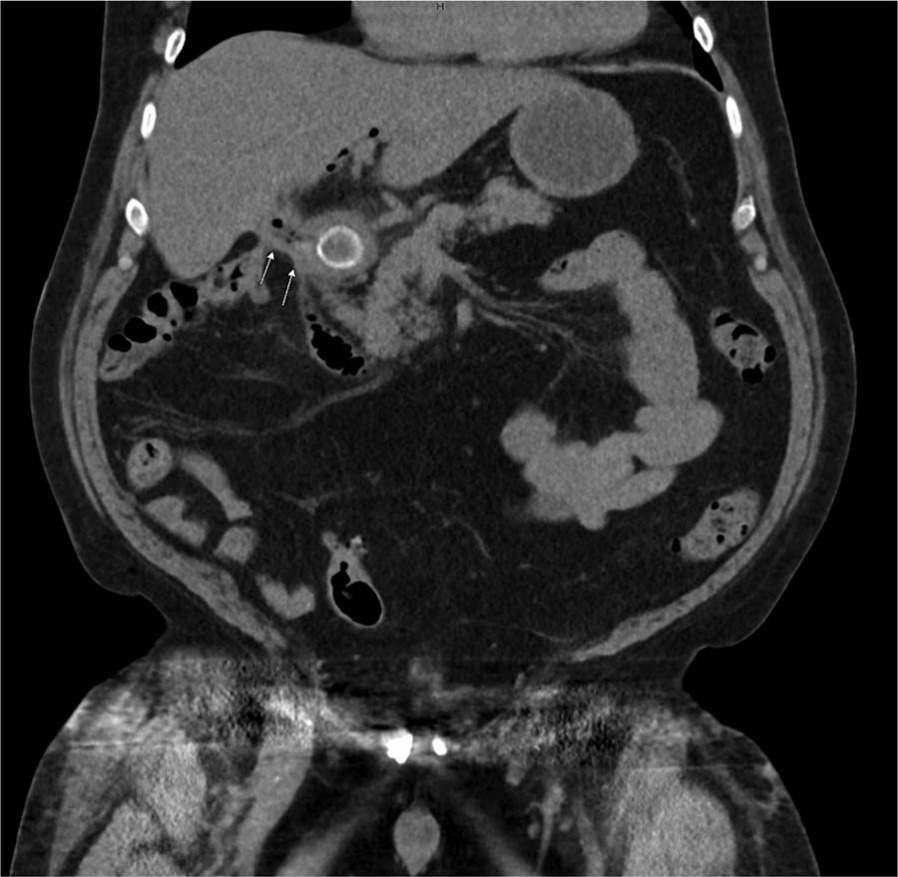
CT of the abdomen without contrast showing a large stone measuring ~3 × 2.1 cm in the proximal duodenum with surrounding induration suggesting a choledochoenteric fistula as indicated by the arrows.

**Figure 2:**
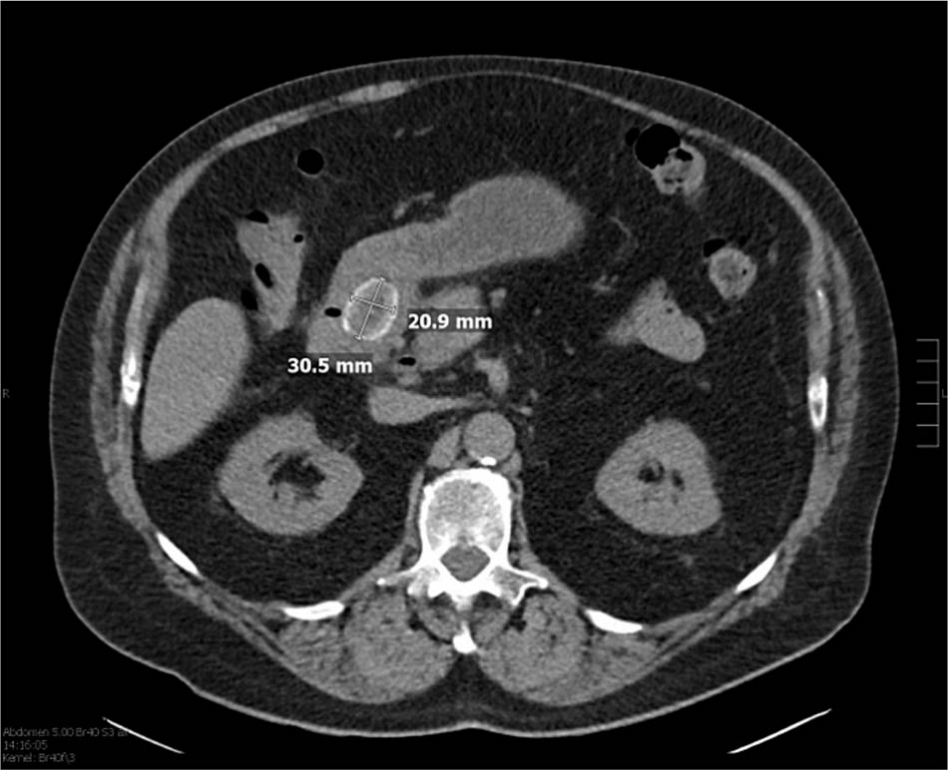
CT of the abdomen without contrast showing biliary gas, suspicious for cholangitis, in addition to the previously mentioned stone.

Laboratory studies showed leukocytosis with a white blood cell count of 22.9 × 10^3^/μl, neutrophils at 88%, and elevated liver function tests: SGPT (ALT) at 483 U/l, SGOT (AST) at 384 U/l, alkaline phosphatase at 198 U/L, and bilirubin at 2.8 mg/dl. An initial ultrasound showed a nonvisualized gallbladder. As shown in Fig. 1, CT abdomen pelvis without contrast identified a large stone in the proximal duodenum (3 × 2.1 cm) with surrounding induration, suggesting a choledochoenteric fistula. Biliary gas indicated cholangitis as shown in Fig. 2.

An NG tube was placed for small bowel decompression on low-intermittent suction due to suspected obstruction, possible perforation, and peritonitis. The patient received cefazolin 2 gm IVPB and metronidazole 500 mg intravenous piggyback, undergoing surgery the day after admission.

During the operation, the stone was palpable in the duodenum, but the liver was densely adherent to the area. Laparoscopic attempts to access the duodenum were unsuccessful, leading to a decision to convert to an open procedure. The gallstone was massaged towards the antrum of the stomach but did not move easily. A gastrostomy at the antrum of the stomach was performed to visualize the impacted gallstone. The stone was ultimately extracted from the posterior antrum after careful manipulation. The gastrostomy was closed primarily with a running suture. Due to difficult anatomy, including an inadequately visualized pylorus and duodenum, two drains were placed: a flat Jackson–Pratt drain along the greater curvature and a Blake drain along the lesser curvature of the repair.

Postoperatively, the patient improved, passing flatus and resolving nausea and vomiting. Liver function tests and leukocytosis decreased. Initially on total parenteral nutrition, the patient transitioned to clear liquids on postoperative day (POD) 4 and to a full liquid diet before discharge. An upper gastrointestinal series on POD 4 showed no gastric outlet obstruction or leaks.

The patient completed a course of empiric metronidazole and ceftriaxone. Pain was well-controlled, and the Jackson–Pratt drain was removed. The decompressive gastrostomy tube and right Jackson–Pratt drain remained in place and were later removed in the outpatient setting.

## Discussion

Bouveret syndrome is a rare condition involving gallstone ileus and a choledochoenteric fistula, presenting significant diagnostic and management challenges. Our patient’s case underscores the importance of a standardized surgical approach, especially when initial endoscopic interventions fail.

Literature presents various surgical options for Bouveret syndrome, including laparoscopic or open gastrotomy, pyloromyotomy, or duodenotomy. The choice often depends on the impacted stone's location and accessibility. A common strategy uses endoscopy to mobilize the stone, followed by an open or laparoscopic procedure for removal [7].

Endoscopic interventions provide a minimally invasive management option, particularly for gallstone removal. However, as illustrated in our case, these techniques can be limited with large or impacted stones. Imaging confirmed gallstone ileus and a suspected choledochoenteric fistula; however, the fistula's extent could only be accurately assessed intraoperatively. This highlights the need for comprehensive evaluation, as endoscopic methods may not always be sufficient for addressing complex cases.

In our case, transitioning from endoscopic management to open surgery became necessary due to the unsuccessful stone removal and the inability to fully evaluate the fistula. This scenario emphasizes that while endoscopic procedures can be effective, they are not always the solution, especially with large stones or complicated fistulas.

Current literature suggests that surgery is necessary in ~90% of cases, with mortality rates ranging from 19% to 24% [8]. Given these statistics, a standardized surgical protocol that encompasses both endoscopic and open techniques is crucial for effective management. Surgeons must carefully assess each case to determine the most appropriate approach, considering factors such as the size and location of the stone, the presence of a fistula, and the patient’s overall health.

This patient’s presentation exemplifies the challenges and considerations involved in managing Bouveret syndrome. While endoscopic techniques offer a less invasive option, their limitations necessitate readiness for more invasive procedures when required. Effective treatment demands a flexible approach, with a focus on minimizing morbidity and mortality through comprehensive management strategies tailored to each patient’s specific needs.

## References

[1] Mavroeidis VK, Matthioudakis DI, Economou NK, et al. Bouveret syndrome—the rarest variant of gallstone ileus: a case report and literature review. Case Rep Surg 2013;2013:1–6. 10.1155/2013/839370. PMC3707284 23864977

[2] Khuwaja S, Azeem A, Semkhayev BA. et al. Bouveret syndrome: when a stone cannot pass the pylorus. ACG Case Rep J 2019;6:e00176. 10.14309/crj.0000000000000176. 31737712 PMC6791613

[3] Ferhatoglu MF . Bouveret’s syndrome: a case based review; clinical presentation, diagnostics and treatment approaches. SiSli Etfal Hastanesi Tip Bulteni 2018;52:28–33. 10.14744/semb.2018.03779. PMC7192252 32377127

[4] Adnan AI, Vaz OP, Lapsia S, et al. Bouveret’s syndrome: a case series and literature review on a gallstone disease causing gastric outlet obstruction. Cureus 2022;14:e27519. 10.7759/cureus.27519. PMC9427024 36060376

[5] Cappell MS, Davis M. Characterization of Bouveret’s syndrome: a comprehensive review of 128 cases. Am J Gastroenterol 2006;101:2139–46. 10.1111/j.1572-0241.2006.00645.x. 16817848

[6] Satchithanandha V, Lau NS, Galevska A, et al. Bouveret syndrome: two approaches one stone. J Surg Case Rep 2023;2023:rjad570. 10.1093/jscr/rjad570. PMC10581706 37854526

[7] Caldwell KM, Lee SJ, Leggett PL, *et al*. Bouveret syndrome: current management strategies. *Clin Exp Gastroenterol* 2018;11:69–75. 10.2147/CEG.S132069. PMC5819584 29497323

[8] Singh G, Merali N, Shirol S, et al. A case report and review of the literature of Bouveret syndrome. Ann Roy Coll Surg Engl 2020;102:e15–9. 10.1308/rcsann.2019.0161. 31859521 PMC6937608

